# Bis(benzylamine) monomers: One-pot preparation and application in dendrimer scaffolds for removing pyrene from aqueous environments

**DOI:** 10.3762/bjoc.9.266

**Published:** 2013-10-31

**Authors:** Olivia N Monaco, Sarah C Tomas, Meghan K Kirrane, Amy M Balija

**Affiliations:** 1 Department of Chemistry, Fordham University, 441 E. Fordham Road, Bronx, NY 10458, USA

**Keywords:** amines, dendrimer, fluorescence studies, imines, pyrene

## Abstract

Bisimine and bisamine AB_2_ monomers have been synthesized from 3,5-diaminobenzoic acid and benzaldehyde derivatives without the need for protective groups or purification. This monomer preparation is universal for various electron-donating and electron-withdrawing benzaldehyde substrates. To demonstrate the versatility of these previously unreported AB_2_ monomers in the formation of high molecular weight structures, novel first-generation dendrimers and hybrid second-generation dendrimers have been synthesized. Using fluorescence spectroscopy, pyrene was shown to be removed from an aqueous environment upon exposure to thin dendrimer films, with the first-generation dendrimer removing 70% of the pyrene within 30 min and the hybrid second-generation dendrimers removing 38–52%. Inclusion formation constants were calculated to be on the order of 10^9^–10^11^ M^−1^ and are comparable to the values of previously reported macromolecules. These results illustrate that size may not influence pyrene removal as effectively as composition.

## Introduction

Highly-branched polymeric systems provide an attractive route for removing pollutants from water due to their interior cavities and their ease of formation [1]. While several promising approaches have been reported [2], these polymeric water-purification systems utilize a patching method, in which the periphery of known architectures is modified with specific functionalities for water treatment processes. Alternatively, dendrimers [3–4] provide the branched architecture while maintaining the ability to incorporate functional groups at precise locations, which may be ideal for removing specific pollutants. However, the use of dendrimers may be hindered without the development of new, more convenient approaches to prepare these systems with minimal purification [5]. Although several strategies offer solutions towards preparing dendrimers more efficiently [6–10], these methods are limited to specific functionalities. There remains an unmet need for novel, flexible synthetic pathways to broaden the functional groups utilized to fine-tune the periphery, core, and branching sites of dendrimers.

The objective of the research disclosed is the development of a straightforward synthesis for novel dendrimers that can effectively remove small organic molecule pollutants from water. Furthermore, the ability to fine-tune the dendrimer easily is desired. The focus of this report is on three areas of progress: (1) the synthesis of new AB_2_ monomers without the use of protective groups and with minimal purification; (2) the incorporation of these monomers into dendrimeric architectures; and (3) the examination of how effectively these new dendrimers remove a representative organic pollutant from an aqueous solution.

## Results and Discussion

Condensation of 3,5-diamino benzoic acid (**1**) with 2.5 equiv of benzaldehyde (**2a**) in methanol at room temperature resulted in the precipitation of bisimine monomer **3a,** which was isolated cleanly by filtration after 20 minutes [11–12]. Electron-deficient, electron-rich, and sterically hindered benzaldehydes **2b**–**g** could also be utilized to prepare the corresponding bisimine products (Table 1, compounds **3b**–**g**). Minimal impact on the overall yield of the reaction was observed upon varying the benzaldehyde concentration or substituting absolute ethanol for methanol; however, elevated temperatures hindered the ability to cleanly obtain bisimines **3**.

**Table 1:** Formation of bisimine compounds **3a**–**g**.

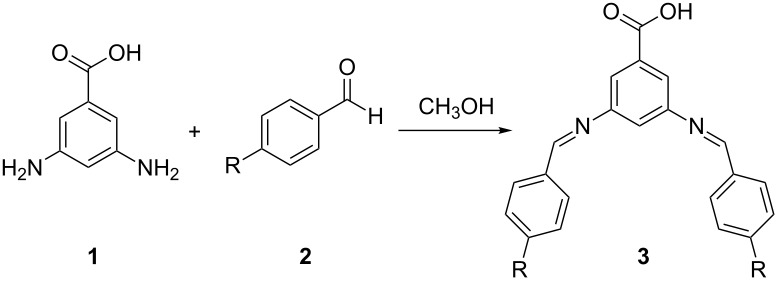				

entry	benzaldehyde	product	R	yield (%)^a^

1	**2a**	**3a**	H	78
2	**2b**	**3b**	OCH_3_	81
3	**2c**	**3c**	CH_3_	72
4	**2d**	**3d**	F^b^	86
5	**2e**	**3e**	Cl^b^	61
6	**2f**	**3f**	NO_2_	93
7	**2g**	**3g**	1-napthaldehyde	78

^a^Isolated yields. ^b^90% pure by ^1^H NMR spectroscopy.

Alternatively, the condensation of 3,5-diamino benzoic acid with 2.1 equiv of benzaldehyde (**2a**) in methanol at room temperature followed by in-situ reduction with NaBH_4_ and acidification with 2 N HCl resulted in the precipitation of bisamine **4a**. Bisamine products were obtained with electron-deficient, electron-rich, and sterically hindered benzaldehydes (Table 2, Compounds **4b**–**h**). The formation of **4f** was difficult, resulting in lower overall yields and longer reaction times, possibly due to the electron-withdrawing properties of the nitro group [13]. Replacing NaBH_4_ with other hydride sources such as NaBH_3_CN did not impact the reduction rate of **4f**. To ensure a clean isolation of bisamines **4**, the benzaldehyde starting material needed to be free from oxidized aldehyde byproducts. Using elevated temperatures to promote the formation of bisamines **4** was unsuccessful. This result was in contrast to comparable systems [14], which required heat to form benzyl amine products exclusively. A preliminary examination using ^1^H NMR spectroscopy suggested that twelve hours were needed to completely reduce the proposed intermediate imine **3**. A mixture of products was obtained when **1** was replaced with methyl 3,5-diaminobenzoate.

**Table 2:** Formation of bisamine compounds **4a**–**h**.

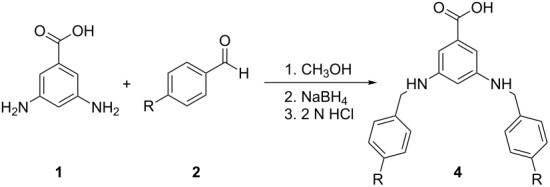				

entry	benzaldehyde	product	R	yield (%)^a^

1	**2a**	**4a**	H	53
2	**2b**	**4b**	OCH_3_	79
3	**2c**	**4c**	CH_3_	35
4	**2d**	**4d**	F	48
5	**2e**	**4e**	Cl	34
6	**2f**	**4f**	Br	50
7	**2g**	**4g**	NO_2_	18
8	**2h**	**4h**	1-napthaldehyde	38

^a^Product precipitated out of solution upon addition of 2 N HCl. The solid was filtered and washed with cold methanol.

The single-pot process of preparing derivatized bisimine and bisamine products from 3,5-diaminobenzoic acid (**1**) and benzaldehyde derivatives eliminates the use of protective groups, removes the need for purification, and follows the general principles of atom economy [15]. This simple one-step approach is in contrast to the synthesis of the oxygen analogue of **4a**, the well-known AB_2_ bis(benzyl) ether monomer, which is prepared in three steps from 3,5-dihydroxybenzoic acid and benzyl bromide. When synthesizing the bis(benzyl) ether monomer, the carboxylic acid group must be masked, and the intermediates must be purified through column chromatography [16]. Furthermore, although many benzyl bromide compounds are commercially available, they are typically lachrymators and are considered hazardous as compared with their benzaldehyde counterparts. Therefore, the bisimine and bisamine synthesis disclosed in this paper are ideal for the generation of functionalized systems with a higher molecular weight without the use of hazardous reagents or generating large quantities of waste as compared with previously published systems.

Bisimine **3a** was stable to ambient conditions in air over four months but degraded to release benzaldehyde within two hours in CHCl_3_, CH_2_Cl_2_, and THF as noted by the red shift in the UV–vis absorption spectrum. ^1^H NMR spectroscopic analysis confirmed the disappearance of the imine peak upon exposure of bisimine **3** to acid. Hydrolysis occurred at different rates with varying functionalities on **3**; however, a quantitative comparison of how the substituents affected hydrolysis could not be adequately obtained. Bisamine **4** was found to be more stable in air although it began to decompose after exposure to organic solvents for one hour.

**Scheme 1:**
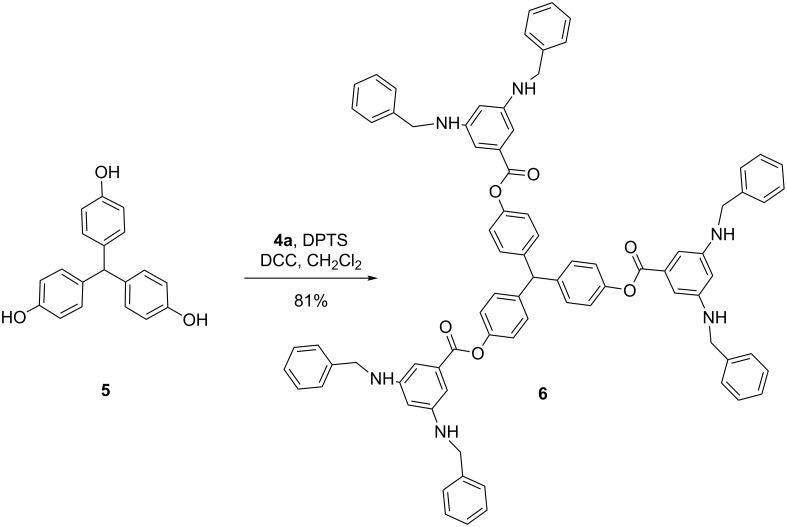
Synthesis of first generation bisamine based dendrimer **6**.

While various applications are possible with monomers **3** and **4**, the AB_2_ structure of these compounds is ideal for incorporation into dendritic systems [17–18]. As a proof of concept, the preparation of first-generation dendrimers was attempted with monomers **3a** and **4a**. Although imine-based dendrimers had been previously reported [19–20], bisimine **3a** readily degraded to its starting materials under the condensation conditions necessary to form the dendrimer. Alternatively, the bisamine scaffold **4** was proposed to be superior to monomer **3** for incorporation into dendrimers due to its improved stability. Condensation of **4a** with triphenol core **5** [16] resulted in the formation of first-generation dendrimer **6** (Scheme 1). Though successfully prepared, the dendrimer decomposed in most organic solvents, making it difficult to purify and analyze. Attempts to prepare other first-generation dendrimers were not pursued due the perceived instability.

**Scheme 2:**
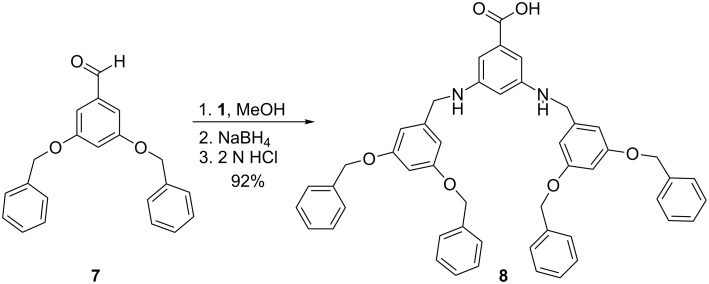
Synthesis of dendron **8**.

To obtain a more stable system, 3,5-bis(benzyloxy)benzaldehyde **7** was designed such that upon condensation with 3,5-diaminobenzoic acid, a hybrid bisamine/benzyl ether product would be obtained. It was proposed that the benzyl ether groups would mask the bisamine functionalities and result in increased compound stability. Structure **7** was successfully prepared through a multi-step synthetic scheme [21]. Condensation of **1** with 2.1 equivalents of benzaldehyde **7** followed by in situ reduction with NaBH_4_ and acidification with 2 N HCl provided dendron **8** (Scheme 2). Compound **8**, itself an AB_2_ monomer similar to compounds **4a**–**4g**, was stable in CDCl_3_ as visualized by ^1^H NMR spectroscopy.

**Scheme 3:**
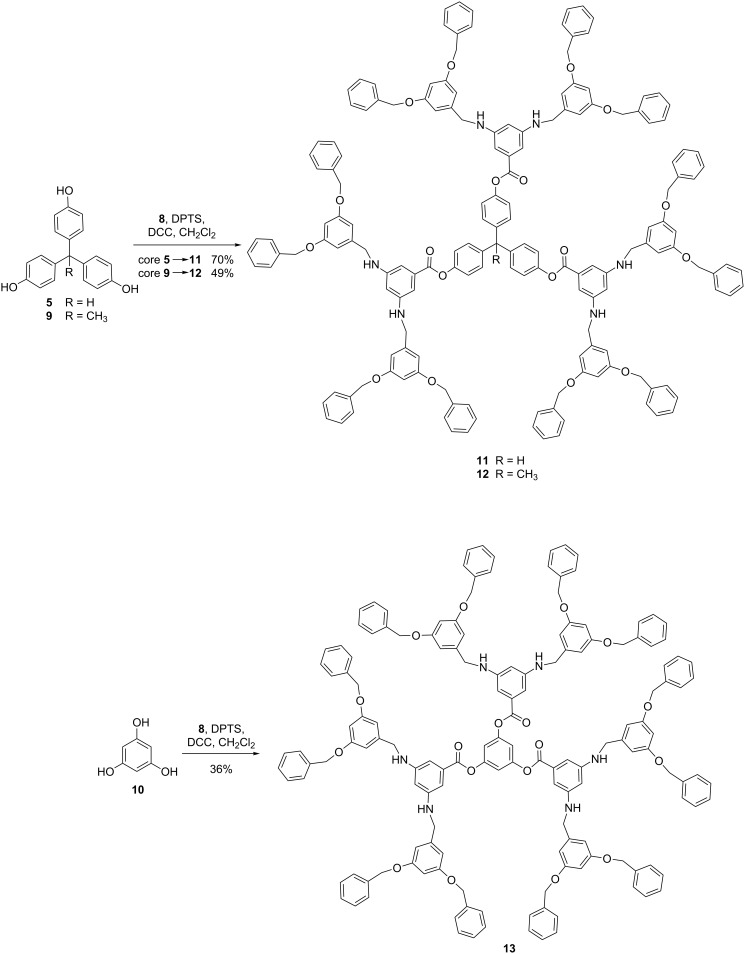
Synthesis of hybrid dendrimers **11**–**13**.

Due to the improved stability of **8** in organic solvents, second-generation dendrimers containing the dendron and triphenol cores **5**, **9**, and **10** (Schemes 1 and 3) were designed. The triphenol cores were utilized to examine how the core diameter and shape influenced the chemical and physical properties of the resulting dendrimers. Cores **5** and **9** were calculated to exist in a helical conformation with an average diameter of 5.7 Å while **10** was present as a planar structure with an average diameter of 2.1 Å [22]. It was proposed that the cores with higher molecular weight would influence the overall properties of the corresponding dendrimer more than the smaller core. Standard coupling conditions [16] of **8** with triphenol cores **5**, **9** and **10** resulted in second-generation dendrimers **11**–**13** (Scheme 3), which were successfully purified by size exclusion chromatography (SEC). The products were determined to be >90% pure through ^1^H NMR spectroscopy and their molecular weights were confirmed by MALDI–TOF. The resulting novel dendrimers contained six amino groups within the interior and twelve ether functionalities along the periphery.

Studies then were performed to determine whether the dendrimers could remain intact upon exposure to various external conditions. To investigate their stability in organic solvents, dendrimers **11**–**13** were separately dissolved in chloroform, and their ^1^H NMR spectra were obtained. While the first-generation dendrimer **6** decomposed within one hour, the second-generation dendrimers **11**–**13** were stable under similar conditions. Additional studies focused on the stability of the dendrimers in water at pH 7. Thin solid films of **6** and **11** were prepared on the bottom of a beaker and water at pH 7 was added to the beaker. After three days, the water was removed and the ^1^H NMR spectra were obtained. No changes in the spectra were observed, which indicated that the dendrimers remained intact in an aqueous solution. Therefore, pure second-generation hybrid dendrimers could be isolated and used in different solvent environments without being degraded.

**Figure 1:**
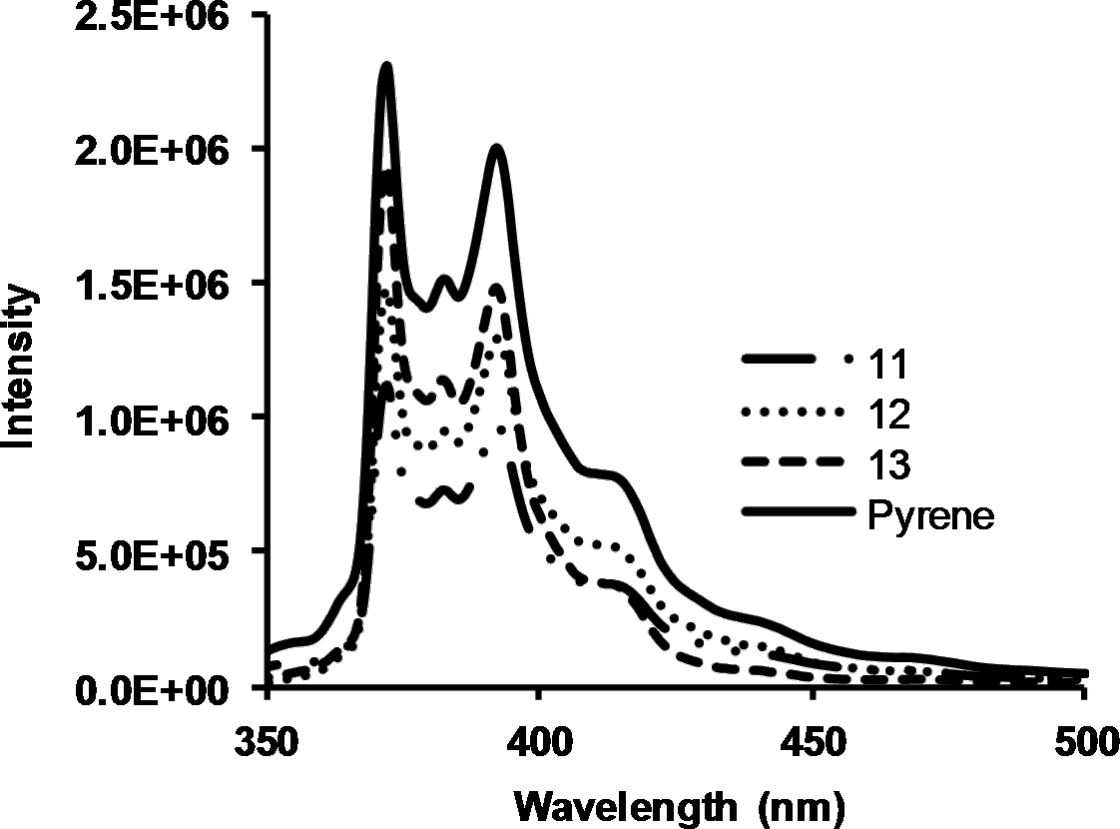
Fluorescence of a saturated aqueous solution of pyrene after 30 min exposure to dendrimers **11**–**13**.

Due to their hydrophobic periphery and larger size, hybrid dendrimers **11**–**13** were envisioned as potential scaffolds to encapsulate small molecule organic pollutants such as polycyclic aromatic hydrocarbons (PAHs) [23–25]. The presence of PAHs in drinking water is a growing concern due to their carcinogenic properties and persistence in the environment [26–27]. Current methods to remove these hydrocarbons are becoming ineffective. To examine the ability of the hybrid dendrimers to remove small organic pollutants, saturated aqueous solutions of pyrene [28], a representative PAH, were introduced to beakers containing thin films of the hydrophobic dendrimers [29–30]. Aliquots were taken at 30 min, 60 min, and two days, and the pyrene fluorescence intensity was determined by fluorescence spectroscopy [31]. After 30 min, a 38–52% decrease in the pyrene fluorescence intensity was recorded with dendrimers **11**–**13** (Figure 1), a change that was not observed in the absence of the dendrimers.

The intensity of the pyrene fluorescence continued to decrease upon longer exposure to the dendrimer films. After two days, dendrimers **11** and **12**, which contained the cores with larger diameters, showed a 95% decrease in pyrene fluorescence intensity, while the exposure to dendrimer **13** resulted in a 70% reduction. No additional decrease in the fluorescence intensity of pyrene was observed over time. As a comparison, the exposure of first-generation dendrimer **6** to a saturated aqueous pyrene solution lead to a 74% decrease in pyrene fluorescence intensity after 30 minutes and 90% decrease in pyrene fluorescence intensity after two days.

This decrease in the pyrene fluorescence intensity upon exposure to the dendrimers may suggest that a hydrophobic, non-specific interaction is occurring between the pyrene and dendrimer film. Over time, the dendrimer film becomes saturated with the pyrene, resulting in its inability to remove additional molecules. Although the size of the dendrimer core did not dramatically influence the pyrene fluorescence signal after 30 min in dendrimer **13**, the smaller-core dendrimer was not as effective over two days. Furthermore, first-generation dendrimer **6** resulted in a higher initial change in the pyrene signal, which is hypothesized to result from the greater density of amine groups, which could favorably interact with the PAHs [32].

Pyrene inclusion formation constants were calculated using the measurement results obtained with the thin film experiments after two days (Table 3). The inclusion values were large, ranging from 10^9^ to 10^11^ M^−1^, and are comparable to previous results obtained with cyclodextrin polymers (10^9^ M^−1^) [33] and alkylated fifth-generation diaminobutane poly(propylene imine) dendrimers (10^8^ M^−1^) [28–30]. The large magnitude is proposed to be due to the thermodynamically favorable process of pyrene moving from a hydrophilic to a hydrophobic environment. A similar conclusion can be drawn from calculating the Gibbs free energies of each system, which lie between −13 and −15 kcal/mol and further demonstrate the favorable interaction of the dendrimers with pyrene in an aqueous environment [29–30]. The capacity of the dendrimers to remove pyrene was estimated to be between 3.12 × 10^−7^ to 6.34 × 10^−7^ mol of pyrene per gram of dendrimer. Current levels of pyrene in industrialized countries range from 1.48 × 10^−12^ to 1.98 × 10^−10^ mol of pyrene per liter of water [34]. Qualitatively, relatively small amounts of dendrimer (<1 g) should effectively remove pyrene from 1 L of stagnant water.

**Table 3:** Inclusion formation constants *K* (M^−1^) and Gibbs free energies Δ*G*° (kcal/mol) of dendrimers **6** and **11**–**13** and pyrene.

entry	compound	*K* (M^−1^)	Δ*G*° (kcal/mol)	capacity (mol pyrene/g dendrimer)

1	**6**	2.44 × 10^10^	−14.1	6.34 × 10^−7^
2	**11**	1.02 × 10^11^	−15.0	3.12 × 10^−7^
3	**12**	1.62 × 10^11^	−15.3	3.31 × 10^−7^
4	**13**	6.09 × 10^9^	−13.3	3.34 × 10^−7^

Overall, the relatively small dendrimers **6** and **11**–**13** gave similar results compared to polymers with higher molecular weight and therefore, large polymeric systems may not be necessary to effectively remove PAHs from water. Furthermore, the flexible preparation of AB_2_ bisamine monomers using different benzaldehyde starting materials allows dendrimer properties to be readily tuned to remove pollutants more effectively. This is more difficult to accomplish with commercially available dendrimers and polymeric systems. Although not applicable for large scale production due to its instability in organic solvents, first-generation dendrimer **6** does illustrate the importance of the bisamine monomer in removing pyrene.

## Conclusion

Described is the synthesis of bisimine and bisamine AB_2_ monomers through a one-step process, which requires no purification and no manipulations of protective groups. Unlike the analogous benzyl ether AB_2_ compounds previously reported, these monomers were formed at room temperature and were filtered and washed to obtain the desired product. The reaction was found to be general for aromatic aldehydes except strong electron-donating benzaldehydes that resulted in unstable imines. An unprecedented first-generation bisamine-based dendrimer **6** was prepared but was found to degrade readily in organic solvents. More stable second-generation dendrimers composed of hybrid dendrons **8** and one of the three tri-phenol cores **5**, **9**, and **10** were synthesized.

One application for these novel structures is the removal of polycyclic aromatic hydrocarbons, such as pyrene, from water. Upon exposure of an aqueous solution of pyrene to the thin dendrimer film, no significant difference in the pyrene fluorescence intensity was observed after 30 min for the hybrid dendrimers **11**–**13**, regardless of the core composition. After two days, the second-generation dendrimers that contain the larger cores **5** and **9** were more efficient in removing pyrene relative to core **10**. While the larger dendrimers were effective at removing pyrene over two days, the first-generation dendrimer **6** extracted more pyrene after 30 min than **11**–**13**. Pyrene inclusion constants for the dendrimers **6** and **11**–**13** were comparable to other polymeric purification systems, suggesting that large polymeric systems are not necessary to remove pyrene. Rather, the composition of the purification system appears to play an important role. Current efforts are focused on incorporating the bisamine dendron motif into additional supramolecular structures such as star polymers and hyperbranched systems and examining how fine-tuning the steric and electronic nature of the bisamine AB_2_ monomers influences the removal of pyrene from water. The results of these studies will be reported in due course.

## Supporting Information

File 1 Full experimental synthetic procedures for compounds **3a**–**g**, **4a**–**h**,**6**–**8**, and **11**–**13**, and pyrene fluorescence spectral data.
