# Supplementary material for: Bis(benzylamine) monomers: One-pot preparation and application in dendrimer scaffolds for removing pyrene from aqueous environments
Source: Beilstein J Org Chem. 2013 Oct 31;9:2320–7. doi: 10.3762/bjoc.9.266 (PMC3869215; doi:10.3762/bjoc.9.266)
Supplement: File 1 — Full experimental synthetic procedures for compounds 3a–g, 4a–h,6–8, and 11–13, and pyrene fluorescence spectral data. [file Beilstein_J_Org_Chem-09-2320-s001.pdf]

Supporting Information

for

Bis(benzylamine) monomers: One-pot preparation and application in dendrimer scaffolds for removing pyrene from aqueous environments

Olivia N. Monaco, Sarah C. Tomas, Meghan K. Kirrane and Amy M. Balija*

Address: Department of Chemistry, Fordham University, 441 E. Fordham Road, Bronx, NY 10458, United States

Email: Amy M. Balija* - balija@fordham.edu

*Corresponding author

Full experimental synthetic procedures for compounds 3a–g, 4a–h,6–8, and 11–13, and pyrene fluorescence spectral data

Table of contents:

I. General experimental

II. Synthesis of bisimine compounds **3**

III. Synthesis of bisamine compounds **4**

IV. Synthesis of dendrimers **6**, **11–13**

V. Thin film fluorescence experiments

VI. Calculations of inclusion formation constants, Gibbs free energies, and dendrimer capacities

VII. Representative ^{1}H and ^{13}C NMR spectral data

VIII. References

I. General experimental

All reactions were performed under an argon gas atmosphere with either flame-dried or oven-dried glassware unless otherwise noted. Reagents were obtained from Aldrich or TCI America. 2-(Dimethylamino)pyridinium *p*-toluenesulfonate (DPTS) was synthesized as reported previously [1]. Solvents and reagents were used without further purification except for the following: MeOH was distilled from $CaSO_4$, CH_2Cl_2 was distilled from CaH_2, benzaldehyde was distilled neat, and phloroglucinol dihydrate was azeotroped 5 times with toluene prior to use. Reactions were monitored by thin layer chromatography (TLC) using silica gel 60 F_{254} glass plates. TLC bands were visualized by UV and phosphomolybdic acid (PMA) stain. Eluent solvent ratios are reported in v/v. Size exclusion chromatography was performed using a 2 cm x 50 cm column of Bio-Rad Bio-Beads S-X1 beads (200–400 mesh) in toluene.

^{1}H NMR spectra were recorded at 300 MHz and ^{13}C NMR spectra were recorded at 75 MHz on a Bruker AV-300 High Performance Digital NMR Spectrometer. Chemical shifts were reported in parts per million (ppm) and coupling constants were reported in Hertz (Hz). ^{1}H NMR spectra obtained in acetone-d_6 were referenced to 2.05 ppm, in $CDCl_3$ were referenced to 7.26 ppm, in DMSO-d_6 were referenced to 2.50 ppm, and in CD_3OD were referenced to 3.30 ppm. ^{13}C NMR spectra obtained in acetone-d_6 were referenced to 29.84 ppm, $CDCl_3$ were referenced to 77.2 ppm, and DMSO-d_6 were referenced to 39.5 ppm. Mass spectra were obtained from University of Illinois Mass Spectrometry Center (Micromass Q-TOF Ultra, ESI) and from New York University (Bruker UltrafleXtreme MALDI-TOF). UV–vis spectra were collected on a Varian Cary 300 Bio UV/Vis spectrophotometer. Fluorescence spectra were obtained on a Jobin Yvon Horiba FluoroMax-3 spectrofluorimeter. Melting points were measured with a Mel-Temp melting point apparatus and are uncorrected.

II. Synthesis of bisimine compounds 3

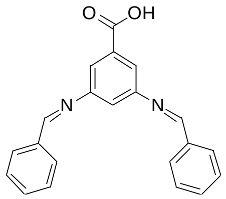

3,5-Bis(benzylideneamino)benzoic acid (3a)

To a heterogeneous solution of 3,5-diaminobenzoic acid (0.50 g, 3.29 mmol) in MeOH (15 mL) was dropwise added benzaldehyde (1 mL, 9.85 mmol). After stirring at room temperature for 25 min, the reaction was filtered and the solid washed with cold MeOH (5 mL) to give 0.84 g (78%) of the desired product. Mp 213-215 ˚C; R_f = 0.84 (CH_2Cl_2:MeOH, 3:1). ^{1}H NMR (acetone-*d*$_6$): δ 8.76 (s, 2H), 8.06-8.02 (m, 4H), 7.78 (d, *J* = 2.0, 2H), 7.57-7.55(m, 6H), 7.41 (t, *J* = 2.0, 1H). ^{13}C NMR (acetone-*d*$_6$): δ 166.9, 162.3, 152.6, 132.2, 131.5, 128.9, 128.9, 119.4, 117.5. HRMS-ESI : *m/z* $[M + H]^+$ calcd for $C_{21}H_{16}N_2O_2$: 329.1290 ; found 329.1302. UV/Vis ($CHCl_3$): λ_{max} 276 nm.

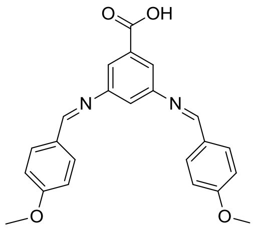

3,5-Bis(4-methoxybenzylideneamino)benzoic acid (3b)

To a heterogeneous solution of 3,5-diaminobenzoic acid (0.50 g, 3.29 mmol) in MeOH (15 mL) was dropwise added *p*-anisaldehyde (1 mL, 8.23 mmol). After stirring at room temperature for 25 min, the reaction was filtered and the solid was washed with cold MeOH (10 mL) to give 1.04 g (81%) of the desired product. Mp 226-229 ˚C; R_f = 0.82 (CH_2Cl_2:MeOH, 3:1). ^{1}H NMR (acetone-*d*$_6$): δ 8.65 (s, 2H), 7.99 (d, *J* = 1.8, 4H), 7.72 (d, *J* = 2.0, 2H), 7.32 (t, *J* = 2.0, 1H), 7.08 (d, *J* = 8.8, 4H), 3.90 (s, 6H). ^{13}C NMR (acetone-*d*$_6$): δ 167.0, 162.2, 161.2, 152.9, 132.7, 130.8, 129.7, 128.8, 119.0, 117.4, 55.4. HRMS-ESI: *m/z* $[M + H]^+$ calcd for $C_{23}H_{20}N_2O_4$ 389.1501; found 389.1493. UV/Vis ($CHCl_3$): λ_{max} 286 nm.

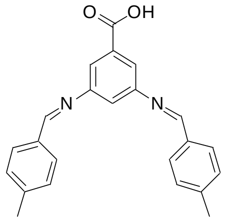

3,5-Bis(4-methylbenzylideneamino)benzoic acid (3c)

To a heterogeneous solution of 3,5-diaminobenzoic acid (0.50 g, 3.29 mmol) in MeOH (15 mL) was dropwise added *p*-tolualdehyde (1.20 mL, 10.2 mmol). After stirring at room temperature for 25 min, the reaction was filtered and the solid washed with cold MeOH (5 mL) to give 0.84 g (72%) of the desired product. Mp 254-256 ˚C; R_f = 0.76 (CH_2Cl_2:MeOH, 3:1). ^{1}H NMR (acetone-d_6): δ 8.69 (s, 2H), 7.93 (d, *J* = 8.1, 4H), 7.75 (d, *J* = 2.0, 2H), 7.35 (m, 5H), 2.42 (s, 6H). ^{13}C NMR (DMSO-d_6): δ 166.9, 162.0, 152.7, 141.9, 133.3, 132.7, 129.4, 128.9, 119.2, 117.4, 21.2. HRMS-ESI: *m/z* $[M + H]^+$ calcd for $C_{23}H_{20}N_2O_2$ 357.1603; found 357.1611. UV/Vis ($CHCl_3$): λ_{max} 270 nm.

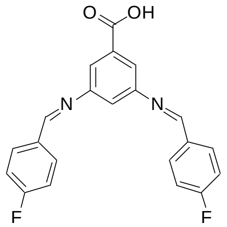

3,5-Bis(4-fluorobenzylideneamino)benzoic acid (3d)

To a heterogeneous solution of 3,5-diaminobenzoic acid (0.50 g, 3.29 mmol) in MeOH (15 mL) was dropwise added 4-fluorobenzaldehyde (1 mL, 9.32 mmol). After stirring at room temperature for 25 min, the reaction was filtered and the solid washed with cold MeOH (5 mL) to give 1.03 g (86%) of the desired product. Mp 223-225 ˚C; R_f = 0.80 (CH_2Cl_2:MeOH, 3:1). ^{1}H NMR (acetone-d_6): δ 8.75 (s, 2H), 8.12-8.08 (m, 4H), 7.77 (d, *J* = 2.0 Hz, 2H), 7.39 (t, *J* = 2.0 Hz, 1H), 7.33 (m, 4H). ^{13}C NMR (DMSO-d_6): δ 167.6, 161.0, 159.1, 149.7, 132.2, 131.0, 130.9, 116.0, 115.7, 108.8. HRMS-ESI: *m/z* $[M + H]^+$ calcd for $C_{21}H_{14}F_2N_2O_2$ 365.1102; found 365.1101. UV/Vis ($CHCl_3$) λ_{max} 267 nm.

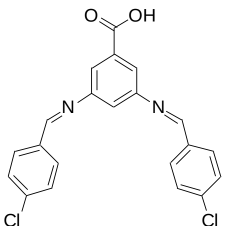

3,5-Bis(4-chlorobenzylideneamino)benzoic acid (3e)

To a heterogeneous solution of 3,5-diaminobenzoic acid (0.50 g, 3.29 mmol) in MeOH (15 mL) was added 4-chlorobenzaldehyde (0.97 g, 6.90 mmol). After stirring at room temperature for 25 min, the reaction was filtered and the solid washed with cold MeOH (5 mL) to give 0.80 g (61%) of the desired product. Mp 242-244 ˚C; R_f = 0.78 (CH_2Cl_2:MeOH, 3:1). ^1H NMR (acetone-d_6): δ 8.77 (s, 2H), 8.07 (d, *J* = 8.5, 4H), 7.79 (d, *J* = 2.0, 4H), 7.60 (d, *J* = 8.4, 4H), 7.43 (t, *J* = 2.1, 1H). ^{13}C NMR (acetone-d_6): δ 166.8, 161.07, 152.2, 136.4, 134.7, 132.8, 130.5, 129.0, 119.6, 117.8. HRMS-ESI: *m/z* $[M + H]^+$ calcd for $C_{21}H_{14}Cl_2N_2O_2$ 397.0511; found 397.0526. UV($CHCl_3$): λ_{max} 266 nm.

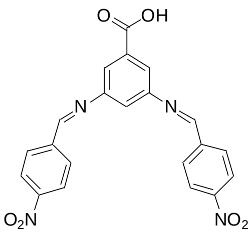

3,5-Bis(4-nitrobenzylideneamino)benzoic acid (3f)

To a heterogeneous solution of 3,5-diaminobenzoic acid (0.50 g, 3.29 mmol) in MeOH (15 mL) was added 4-nitrobenzaldehyde (1.00 g, 6.61 mmol). After stirring at room temperature for 25 min, the reaction was filtered and the solid washed with cold MeOH (5 mL) to give 1.28 g (93%) of the desired product. Mp 259-260 ˚C; R_f = 0.66 (CH_2Cl_2:MeOH, 3:1). ^1H NMR (acetone-d_6): δ 8.97 (s, 2H), 8.44 (d, *J* = 8.8, 4H), 8.33 (d, *J* = 8.9, 4H), 7.91 (d, *J* = 2.0, 2H), 7.57 (t, *J* = 1.9, 1H). ^{13}C NMR (acetone-d_6): δ 166.7, 160.8, 151.8, 149.1, 133.0, 129.9, 124.1, 120.2, 118.3. HRMS-ESI: *m/z* $[M + H]^+$ calcd for $C_{21}H_{14}N_4O_6$ 419.0992; found 419.0995. UV/Vis ($CHCl_3$) λ_{max} 269 nm.

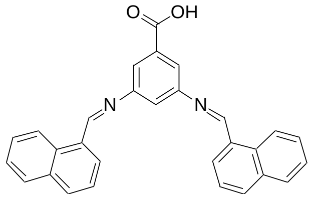

3,5-Bis[(naphthalene-1-ylmethylene)amino]benzoic acid (3g)

To a heterogeneous solution of 3,5-diaminobenzoic acid (0.5 g, 3.29 mmol) in MeOH (15 mL) was dropwise added 1-naphthaldehyde (1.3 mL, 9.57 mmol). After stirring at room temperature for 25 min, the reaction was filtered and washed with cold MeOH (5 mL) to give 1.10 g (78%) of the desired product. Mp 249-251 ˚C; R_f = 0.84 (CH_2Cl_2:MeOH, 3:1). ^{1}H NMR (DMSO-d_6): δ 9.38 (s, 2H), 9.34 (d, J = 8.5, 2H), 8.27 (d, J = 6.3, 2H), 8.17 (d, J = 8.2, 2H), 7.81 (d, J = 7.2, 2H), 7.73-7.647 (m, 7H). ^{13}C NMR (DMSO-d_6): δ 167.0, 162.2, 153.0, 133.5, 132.3, 131.0, 130.9, 130.9, 128.7, 127.7, 126.4, 125.5, 124.8, 119.9, 117.2. HRMS-ESI: *m/z* $[M + H]^+$ calcd for $C_{29}H_{20}N_2O_2$ 429.1603; found 429.1605. UV/Vis ($CHCl_3$): λ_{max} 338 nm.

III. Synthesis of bisamine compounds 4

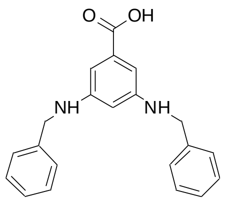

3,5-Bis(benzylamino)benzoic acid (4a)

To a heterogeneous solution of 3,5-diaminobenzoic acid (0.50 g, 3.29 mmol) in MeOH (10 mL) was dropwise added benzaldehyde (0.70 mL, 6.89 mmol). After stirring at room temperature for 20 min, the reaction flask was cooled in an ice water bath and $NaBH_4$ (0.20 g, 5.55 mmol) was added slowly. The reaction mixture was then stirred at room temperature overnight. After cooling the reaction in an ice water bath, distilled water (3 mL) was added and the reaction mixture was acidified with 2N HCl until the product precipitated. The resulting product was filtered and washed with cold distilled water to give 0.58 g (53%) of the desired product. Mp 159-161 ˚C; R_f = 0.80 (CH_2Cl_2:MeOH, 3:1). ^{1}H NMR (acetone-d_6): δ 7.37-7.27 (m, 8H), 7.24-7.18 (m, 2H), 6.70 (d, *J* = 2.1, 2H), 6.20 (t, *J* = 2.2, 1H), 4.30 (s, 4H). ^{13}C NMR (acetone-d_6) δ 168.7, 150.4, 141.0, 132.3, 129.0, 127.9, 127.4, 104.2, 101.4, 47.9. HRMS-ESI: *m/z* $[M + H]^+$ calcd for $C_{21}H_{20}N_2O_2$ 333.1603; found 333.1595. UV/Vis (CH_2Cl_2): λ_{max} 352 nm.

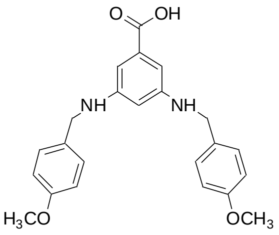

3,5-Bis(4-methoxybenzylamino)benzoic acid (4b)

To a heterogeneous solution of 3,5-diaminobenzoic acid (0.25 g, 1.64 mmol) in MeOH (5 mL) was dropwise added *p*-anisaldehyde (0.43 mL, 3.53 mmol). After stirring at room temperature for 20 min, the reaction flask was cooled in an ice water bath and $NaBH_4$ (0.12 g, 3.17 mmol) was added slowly. The reaction mixture was then stirred at room temperature overnight. After cooling the reaction in an ice water bath, distilled water (3

mL) was added and the reaction mixture was acidified with 2N HCl until the product precipitated. The resulting product was filtered and washed with cold distilled water to give 0.51 g (79%) of the desired product. Mp 152-154 ˚C; R_f = 0.80 (CH_2Cl_2:MeOH, 3:1). ^{1}H NMR (acetone-d_6): δ 7.27 (d, *J* = 8.7, 4H), 6.90 (d, *J* = 8.7, 4H), 6.71 (d, *J* = 2.1, 2H), 6.20 (t, *J* = 2.1, 1H), 4.22 (s, 4H), 3.76 (s, 6H). ^{13}C NMR (acetone-d_6): δ 168.8, 159.6, 150.6, 133.0, 132.6, 129.4, 114.6, 104.4, 101.6, 55.5, 47.6. HRMS-ESI: *m/z* [M + H]$^+$ calcd for $C_{23}H_{24}N_2O_4$ 393.1814; found 393.1813. UV/Vis (CH_2Cl_2): λ_{max} 352 nm.

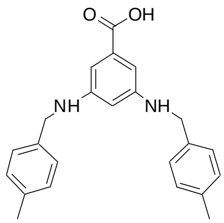

3,5-Bis(4-methylbenzylamino)benzoic acid 4c

To a heterogeneous solution of 3,5-diaminobenzoic acid (0.50 g, 3.29 mmol) in MeOH (5 mL) was dropwise added *p*-tolualdehyde (0.78 mL, 6.62 mmol). After stirring at room temperature for 20 min, the reaction flask was cooled with an ice water bath and $NaBH_4$ (0.20 g, 5.29 mmol) was added slowly. The reaction mixture was then stirred at room temperature overnight. After cooling the reaction in an ice water bath, distilled water (3 mL) was added and the reaction mixture was acidified with 2N HCl until the product precipitated. The resulting product was filtered and washed with cold distilled water to give 0.42 g (35%) of the desired product. Mp 155-157 ˚C; R_f = 0.81 (CH_2Cl_2:MeOH, 3:1). ^{1}H NMR (acetone-d_6): δ 7.18 (d, *J* = 8.0, 4H), 7.16 (d, *J* = 8.0, 4H), 6.69 (d, *J* = 2.1, 2H), 6.19 (t, *J* = 2.1, 1H), 4.23 (s, 4H), 2.28 (s, 6H). ^{13}C NMR (acetone-d_6): δ 169.0, 150.5, 138.1, 136.8, 132.8, 129.7, 128.0, 104.3, 101.4, 47.8, 21.0. HRMS-ESI: *m/z* [M + H]$^+$ calcd for $C_{23}H_{24}N_2O_2$ 361.1916; found 361.1921. UV/Vis (CH_2Cl_2): λ_{max} 353 nm.

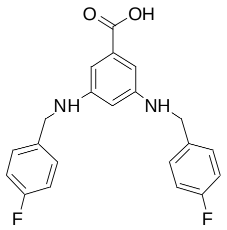

3,5-Bis(4-fluorobenzylamino)benzoic acid (4d)

To a heterogeneous solution of 3,5-diaminobenzoic acid (0.25 g, 1.64 mmol) in MeOH (5 mL) was dropwise added 4-fluorobenzaldehyde (0.43 mL, 4.00 mmol). After stirring at room temperature for 20 min, the reaction flask was cooled with an ice water bath and $NaBH_4$ (0.12 g, 3.17 mmol) was added slowly. The reaction mixture was then stirred at room temperature overnight. After cooling the reaction in an ice water bath, distilled water (3 mL) was added and the reaction mixture was acidified with 2N HCl until the product precipitated. The resulting product was filtered and washed with cold distilled water to give 0.29 g (48%) of the desired product. Mp 145-146 ˚C; R_f = 0.80 (CH_2Cl_2:MeOH, 3:1). ^{1}H NMR (acetone-d_6): δ 7.39-7.34 (m, 4H), 7.08-7.02 (m, 4H), 6.70 (d, J = 2.1, 2H), 6.15 (t, J = 2.1, 1H), 4.29 (s, 4H). ^{13}C NMR (acetone-d_6): δ 168.6, 164.3, 150.5, 137.3, 132.6, 129.9, 104.5, 101.5, 47.4. HRMS-ESI: *m/z* $[M + H]^+$ calcd for $C_{21}H_{18}F_2N_2O_2$ 369.1415; found 369.1417. UV/Vis (CH_2Cl_2): λ_{max} 349 nm.

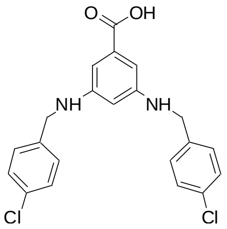

3,5-Bis(4-chlorobenzylamino)benzoic acid (4e)

To a heterogeneous solution of 3,5-diaminobenzoic acid (0.25 g, 1.64 mmol) in MeOH (16 mL) was added 4-chlorobenzaldehyde (0.46 g, 3.30 mmol). After stirring at room temperature for 20 min, the reaction flask was cooled with an ice water bath and $NaBH_4$ (0.12 g, 3.15 mmol) was added slowly. The reaction mixture was then stirred at room temperature overnight. After cooling the reaction in an ice water bath, distilled water (3 mL) was added and the reaction mixture was acidified with 2N HCl until the product

precipitated. The resulting product was filtered and washed with cold distilled water to give 0.22 g (34%) of the desired product. Mp: 155-157 ˚C; R_f = 0.67 (CH_2Cl_2:MeOH, 3:1). ^{1}H NMR (acetone-d_6): δ 7.36-7.29 (m, 8H), 6.68 (d, *J* = 2.1, 2H), 6.12 (t, *J* = 2.1, 1H), 4.31 (s, 4H). ^{13}C NMR (acetone-d_6): δ 168.5, 150.4, 140.3, 132.7, 132.6, 129.7, 104.6, 101.4, 47.4. HRMS-ESI: *m/z* $[M + H]^+$ calcd for $C_{21}H_{18}Cl_2N_2O_2$ 401.0824; found 401.0818. UV/Vis (CH_2Cl_2): λ_{max} 352 nm.

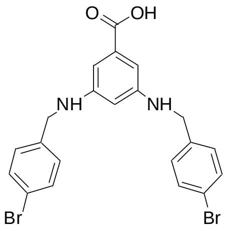

3,5-Bis(4-bromobenzylamino)benzoic acid (4f)

To a heterogeneous solution of 3,5-diaminobenzoic acid (0.25 g, 1.64 mmol) in MeOH (15 mL) was added 4-bromobenzaldehyde (0.65 g, 3.52 mmol). After stirring at room temperature for 20 min, the reaction flask was cooled with an ice water bath and $NaBH_4$ (0.13 g, 3.49 mmol) was added slowly. The reaction mixture was then stirred at room temperature overnight. After cooling the reaction in an ice water bath, distilled water (3 mL) was added and the reaction mixture acidified with 2N HCl until the product precipitated. The resulting product was filtered and washed with cold distilled water to give 0.40 g (50%) of the desired product. Mp: 144-145 ˚C; R_f = 0.80 (CH_2Cl_2:MeOH, 3:1). ^{1}H NMR (acetone-d_6): δ 7.47 (d, *J* = 8.2, 4H), 7.30 (d, *J* = 8.1, 4H), 6.69 (d, *J* = 1.5, 2H), 6.12 (t, *J* = 1.6, 1H), 4.30 (s, 4H). ^{13}C NMR (acetone-d_6): δ 168.5, 150.3, 140.7, 132.5, 132.1, 130.0, 120.7, 140.5, 101.3, 47.3. HRMS-ESI: *m/z* $[M + H]^+$ calcd for $C_{21}H_{18}Br_2N_2O_2$ 488.9813; found 488.9825. UV/Vis (CH_2Cl_2): λ_{max} 351 nm.

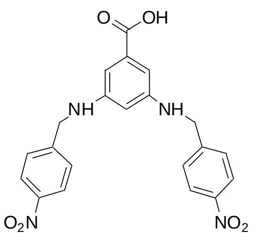

3,5-Bis(4-nitrobenzylamino)benzoic acid (4g)

To a heterogeneous solution of 3,5-diaminobenzoic acid (0.17 g, 1.10 mmol) in MeOH (10 mL) was added 4-nitrobenzaldehyde (0.33 g, 2.21 mmol). After stirring at room temperature for 20 min, the reaction flask was cooled in an ice water bath and $NaBH_4$ (0.14 g, 3.76 mmol) was added slowly. The reaction was then allowed to stir at room temperature overnight. After cooling the reaction in an ice water bath, distilled water (3 mL) was added and the reaction mixture acidified with 2N HCl until the product precipitated. The resulting solid was filtered and washed with cold distilled water. The reduction was performed two additional times with similar amounts of $NaBH_4$ for complete reduction of the imine to obtain 0.08 g (18%) of the desired product. Mp: 140-143 ˚C; R_f = 0.76 (CH_2Cl_2:MeOH, 3:1). ^1H NMR (acetone-d_6): δ 8.16 (d, J = 8.8, 4H), 7.60 (d, J = 8.8, 4H), 6.69 (d, J = 2.1, 2H), 6.05 (t, J = 2.1, 1H), 4.48 (s, 4H). ^{13}C NMR (acetone-d_6): δ 168.8, 150.6, 150.1, 148.3, 133.4, 129.2, 124.7, 105.2, 101.7, 47.9. HRMS-ESI: m/z $[M + H]^+$ calcd for $C_{21}H_{18}N_4O_6$ 423.1305; found 423.1307. UV/Vis (CH_2Cl_2): λ_{max} 347 nm.

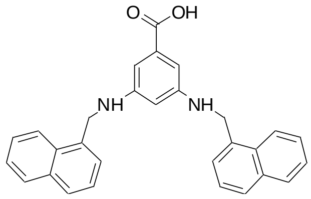

3,5-Bis[(naphthalene-1-yl)amino]benzoic acid (4h)

To a heterogeneous solution of 3,5-diaminobenzoic acid (0.25 g, 1.64 mmol) in MeOH (5 mL) was dropwise added 1-naphthaldehyde (0.48 mL, 3.53 mmol). After stirring at room temperature for 20 min, the reaction flask was cooled in an ice water bath and

$NaBH_4$ (0.12 g, 3.17 mmol) was added slowly. The reaction was then allowed to stir at room temperature overnight. After cooling the reaction in an ice water bath, distilled water (3 mL) was added and the reaction mixture was acidified with 2N HCl until the product precipitated. The resulting product was filtered and washed with cold distilled water to give 0.27 g (38%) of the desired product. Mp: 168-169 ˚C; R_f = 0.88 (CH_2Cl_2:MeOH, 3:1). ^{1}H NMR (acetone-d_6): δ 8.12 (d, *J* = 7.1, 2H), 7.94 (d, *J* = 6.4, 2H), 7.81 (d, *J* = 7.7, 2H), 7.55-7.37 (m, 8H), 6.58 (s, 2H), 4.60 (s, 4H). ^{13}C NMR (acetone-d_6): δ 168.4, 149.7, 135.1, 133.4, 131.8, 131.1, 128.5, 127.2, 126.0, 125.7, 125.4, 124.8, 123.5, 102.2, 99.8, 44.7. HRMS-ESI: *m/z* $[M + H]^+$ calcd for $C_{29}H_{24}N_2O_2$ 433.1916; found 433.1912. UV/Vis (CH_2Cl_2): λ_{max} 349 nm.

IV. Synthesis of dendrimers 11–13

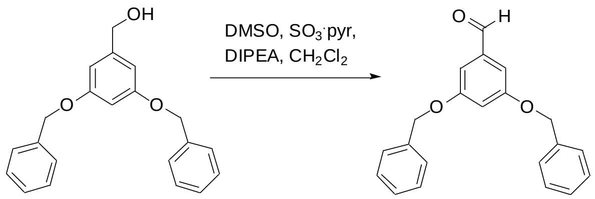

3,5-Bis(benzyloxy)benzaldehyde (7)

To a solution of 3,5-bis(benzyloxy)benzyl alcohol [2] (9.9 g, 31.0 mmol) in CH_2Cl_2 (140 mL) was added DMSO (22 mL, 0.31 mol) and diisopropylethylamine (26.92 mL, 0.154 mol). The mixture was cooled to 0 ˚C and SO_3•pyridine (14.76 g, 92.73 mmol) added. The reaction stirred at 0 °C for one hour, after which it was quenched with saturated aqueous $Na_2S_2O_3$ (310 mL). The solution was extracted with ethyl acetate (3 x 200 mL) and the organic layers combined and dried over $MgSO_4$. Evaporation of the solvent afforded an oil which was purified by silica gel column chromatography in CH_2Cl_2 to give 9.2 g (93%) of the desired product. The spectra was similar to data reported previously [3].

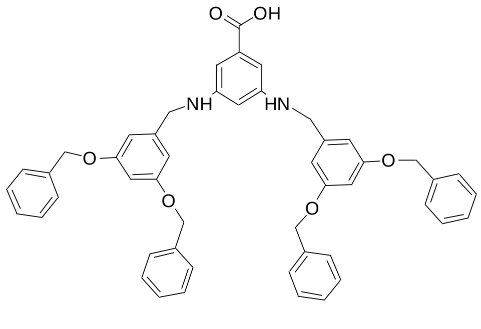

Hybrid dendron 8

To a heterogeneous solution of 3,5-diamino benzoic acid (0.23 g, 1.50 mmol) in MeOH (2 mL) was added a solution of aldehyde **7** (1.00 g, 3.15 mmol) in MeOH (20 mL). After stirring at room temperature for 20 min, the reaction flask was cooled in an ice bath and $NaBH_4$ (0.096 g, 2.53 mmol) was added. The reaction was allowed to stir at room temperature overnight, after which TLC in 5% MeOH, 1% Et_3N in CH_2Cl_2 confirmed its completion. The reaction was cooled to 0 °C and distilled H_2O (5 mL) was added. The

solution was neutralized with 2N HCl (6.4 mL) and extracted with CH_2Cl_2 (3X, 30 mL). The organic layers were combined and dried over $MgSO_4$ and rotary evaporated to afford a green foam. The crude product was purified by silica gel column in 5% MeOH and 1% Et_3N in CH_2Cl_2 to afford 0.98 g (92%) of a gold foam product. R_f = 0.45 (5% MeOH in CH_2Cl_2 with 1% Et_3N). ^1H NMR ($CDCl_3$): δ (ppm): δ 7.42-7.28 (m, 20H), 6.84 (d, *J* = 2.0, 2H), 6.62 (d, *J* = 2.2, 4H), 6.50 (t, *J* = 2.2, 2H), 5.94 (t, *J* = 2.5, 1H), 4.99 (s, 8H), 4.24 (s, 4H). ^{13}C NMR ($CDCl_3$): δ (ppm) 172.3, 160.0, 148.8, 142.2, 136.7, 136.6, 128.4, 127.8, 127.4, 106.3, 104.5, 100.7, 99.9, 69.8, 44.6. HRMS-ESI: *m/z* $[M + H]^+$ calcd for $C_{49}H_{44}N_2O_6$ 757.3277 ; found 757.3278. UV/Vis (CH_2Cl_2) λ_{max} 348 nm.

Hybrid dendrimer 11

To a solution of monomer **8** (0.25 g, 0.33 mmol) in CH_2Cl_2 (5 mL) was added DPTS (0.11 g, 0.36 mmol) and 4,4',4''-trihydroxytriphenylmethane (**5**) (0.31 g, 0.11 mmol). After 15 min of stirring at room temperature, DCC (0.75 g, 0.36 mmol) was added. The reaction stirred for an additional 15 min before being filtered through a pad of silica gel with CH_2Cl_2 with 1% Et_3N and the solvent evaporated. The crude product was purified by size exclusion chromatography in toluene to obtain 0.19 g (70%) of the dendrimer. R_f = 0.63 (CH_2Cl_2 with 1% Et_3N); ^1H NMR ($CDCl_3$): δ (ppm) 7.39-7.29 (m, 60H), 7.12 (app quart, *J* = 6.7, 12H), 6.87 (d, *J* = 2.0, 6H), 6.61 (d, *J* = 2.2, 12H), 6.53 (t, *J* = 2.2, 6H), 6.03 (t, *J* = 2.0, 3H), 5.50 (s, 1H), 5.00 (s, 24H), 4.24 (s, 12H), 4.10 (broad s, 6H). ^{13}C NMR ($CDCl_3$): δ (ppm) 160.2, 149.1, 141.7, 136.8, 131.0, 130.4, 128.6, 128.0, 127.5, 121.1, 106.4, 104.6, 100.9, 70.14, 48.38. MS (MALDI) dithranol *m/z* 2508.8 (M^+).

Hybrid dendrimer 12

To a solution of monomer **8** (0.25 g, 0.33 mmol) in CH_2Cl_2 (5 mL) was added DPTS (0.12 g, 0.36 mmol) and 4,4',4''-trihydroxytriphenylethane (**9**) (0.03 g, 0.10 mmol). After 15 min of stirring at room temperature, DCC (0.08 g, 0.36 mmol) was added. The reaction was stirred an additional 15 min before removal of the solvent *in vacuo* to give a yellow foam. Purification of the crude product by silica gel column chromatography (CH_2Cl_2 with 1% Et_3N → CH_2Cl_2 with 5% MeOH and 1% Et_3N) followed by size exclusion chromatography in toluene afforded 0.12 g (49%) of the dendrimer. R_f = 0.74

(5% MeOH in CH_2Cl_2 with 1% NEt_3). ^1H NMR ($CDCl_3$): δ (ppm) = 7.46-7.30 (m, 60H), 7.13 (app quart, *J* = 8.7, 12H), 6.88 (d, *J* = 2.0, 6H), 6.62 (d, *J* = 2.2, 12H), 6.52 (t, *J* = 2.2, 6H), 6.05 (t, *J* = 1.9, 3H), 5.10 (s, 3H), 5.00 (s, 24H), 4.24 (s, 12H), 4.10 (broad s, 6H), 2.19(s, 3H). ^{13}C NMR ($CDCl_3$): δ (ppm) = 165.7, 160.2, 149.3, 149.2, 146.1, 141.7, 136.8, 131.1, 129.7, 128.5, 128.0, 127.5, 121.1, 108.3, 106.5, 104.6, 101.0, 70.1, 51.7, 48.4. MS (MALDI) dithranol *m/z* 1785.6 (M^+ - 738.4 + H^+).

Hybrid dendrimer 13

To a solution of monomer **7** (0.25 g, 0.33 mmol) in CH_2Cl_2 (5 mL) was added DPTS (0.11 g, 0.36 mmol) and phloroglucinol (**10**) (0.01 g, 0.11 mmol). After 15 min of stirring at room temperature, DCC (0.08 g, 0.36 mmol) was added. The reaction stirred for an additional 15 min before being filtered through a pad of silica gel with CH_2Cl_2 with 1% Et_3N and the solvent evaporated. The crude product was purified by size exclusion chromatography in toluene to obtain 0.09 g (36%) of the dendrimer. R_f = 0.73 (CH_2Cl_2 with 1% Et_3N); ^1H NMR ($CDCl_3$): δ (ppm) 7.40-7.29 (m, 60H), 7.06 (s, 3H), 6.84 (d, *J* = 2.0, 6H), 6.61 (d, *J* = 2.2, 12H), 6.52 (t, *J* = 2.2, 6H), 6.02 (t, *J* = 1.9, 3H), 4.99 (s, 24H), 4.24 (s, 12H), 4.10 (broad s, 6H). ^{13}C NMR ($CDCl_3$): δ (ppm) 160.3, 149.3, 141.7, 136.9, 130.0, 128.6, 128.0, 127.6, 106.5, 104.6, 101.1, 70.1, 48.4. MS (MALDI) dithranol *m/z* 2346.1 (M^+ + $4H^+$).

Dendrimer 6

To a solution of 0.250 g (0.75 mmol) of **4a** in 5 mL of CH_2Cl_2 was added 0.07g (0.23 mmol) of 4,4',4''-trihydroxytriphenylmethane (**5**) and 0.22 g (0.79 mmol) of DPTS. After stirring at room temperature for 15 min, 0.17 g (0.83 mmol) of DCC was added. The reaction was stirred for 15 min, filtered and the filtrate was removed in vacuo. The crude product was purified by flash column chromatography (pet ether→CH_2Cl_2 with 1% Et_3N) and by size exclusion chromatography in THF to give 0.20 g (81%) of the desired dendrimer. ^1H NMR ($CDCl_3$): δ (ppm) 7.34 (d, *J* = 4.4, 22H), 7.31-7.28 (m, 8H), 7.13 (app quart, *J* = 8.78, 12H), 6.89 (d, *J* = 2.1, 6H), 6.08 (t, *J* = 2.0, 3H), 5.59 (s, 1H), 4.31 (s, 12H), 4.12 (broad s, 6H). ^{13}C NMR ($CDCl_3$): δ (ppm) 165.8, 149.2, 139.0, 130.5,

129.8, 129.0, 128.7, 127.5, 127.3, 121.6, 104.5, 48.3. HRMS-ESI: *m/z* $[M + H]^+$ calcd for $C_{82}H_{71}N_6O_6$ 1235.5435; found 1235.5431.

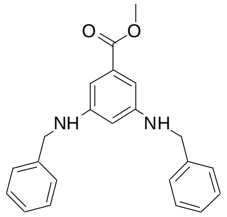

Methyl 3,5-bis(benzylamino)benzoate (14)

To a solution of methyl 3,5-diaminobenzoate (0.5 g, 3.01 mmol) in MeOH (10 mL) was added benzaldehyde (0.61 mL, 6.02 mmol). The solution stirred at room temperature for 20 min, after which the flask was cooled to 0 ˚C before adding $NaBH_4$ (0.57g, 15.05 mmol) in three portions. After stirring overnight at room temperature, the flask was cooled to 0 ˚C and water slowly added. A brown solid precipitated which was filtered and washed with ice-cold MeOH. The product was purified by silica gel column chromatography in 10:1 CH_2Cl_2:MeOH to afford 0.096 g (9%) of the desired product . R_f = 0.96 (10:1 CH_2Cl_2:MeOH; ^1H NMR ($CDCl_3$): δ (ppm).34-7.28 (m, 10H), 6.75 (d, *J* = 2.1, 2H), 6.03 (t, *J* = 2.1, 1H), 4.29 (s, 4H), 4.04 (broad s, 2H), 3.85 (s, 3H). ^{13}C NMR ($CDCl_3$): δ (ppm) 167.7, 149.2, 139.2, 131.8, 128.7, 127.5, 127.3, 104.1, 101.0, 52.0, 48.3. HRMS-ESI: *m/z* $[M + H]^+$ calcd for $C_{22}H_{23}N_2O_2$ 347.1760 ; found 347.1760.

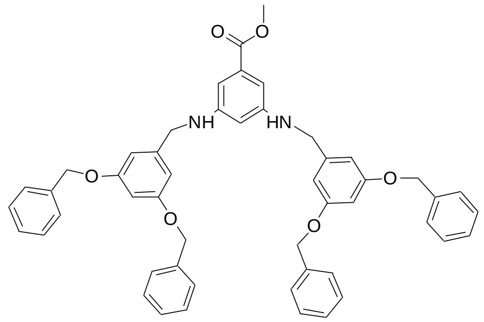

Hybrid dendron 15

A solution of **8** (0.10 g, 0.13 mmol) in MeOH (5 mL) and sulfuric acid (0.1 mL) was refluxed at 65 °C overnight. After the reaction was cooled to room temperature, the

solvent was evaporated to afford a brown solid, which was dissolved in ethyl acetate (5 mL) and extracted with saturated $NaHCO_3$ (3 x 2 mL), distilled H_2O (2 x 2 mL), and brine (2 x 2 mL). The organic layer was dried over $MgSO_4$ and evaporated to afford 0.07 g (65%) of a brown film. R_f = 0.96 (5% MeOH in CH_2Cl_2 with 1% Et_3N); ^1H NMR ($CDCl_3$): δ (ppm) 7.41-7.29 (m, 20H), 6.73 (d, *J* = 2.1, 2H), 6.60 (d, *J* = 2.2, 4H), 6.45 (t, *J* = 2.2, 2H), 5.98 (t, *J* = 2.1, 1H), 5.00 (s, 8H), 4.23 (s, 4H), 3.84 (s, 3H). ^{13}C NMR ($CDCl_3$): δ (ppm) 167.9, 160.2, 149.2, 141.9, 136.9, 131.8, 128.6, 128.1, 127.7, 106.5, 104.1, 101.2, 101.0, 70.1, 52.1, 48.4. HRMS-ESI: *m/z* $[M + H]^+$ calcd for $C_{50}H_{47}N_2O_6$ 771.3423 ; found 771.3434.

V. Thin film fluorescence experiments

Preparation of dendrimer films

Films were created by dissolving 0.02 mmol of dendrimer or 0.06 mmol of dendron in 5 mL of CH_2Cl_2 in a 100 mL beaker and then removing the solvent with a stream of air. Films were protected from dust and other contaminants by placing parafilm over the opening.

Preparation of saturated aqueous pyrene standard

A 1 mL aliquot of a 3.7 x 10^{-2} M solution of pyrene in CH_2Cl_2 was added to a 250 mL volumetric flask. The solvent was evaporated and distilled water was added. The flask was then sonicated and allowed to sit for 1 d prior to use.

Encapsulation study procedures

An aliquot of saturated aqueous pyrene standard prepared above was filtered through a cotton plug into a cuvette. The cuvette was excited at a wavelength of 325 nm and the fluorescence emission spectrum obtained from 340–500 nm.

To each beaker containing a film was added 25 mL of filtered saturated aqueous pyrene. After time points of 30 min, 60 min, and 2 d, an aliquot was removed, filtered, and its fluorescence measured. Two studies were performed for each sample to ensure consistency. As a control, the films were exposed to distilled water and the fluorescence obtained. The data was analyzed and graphed as shown.

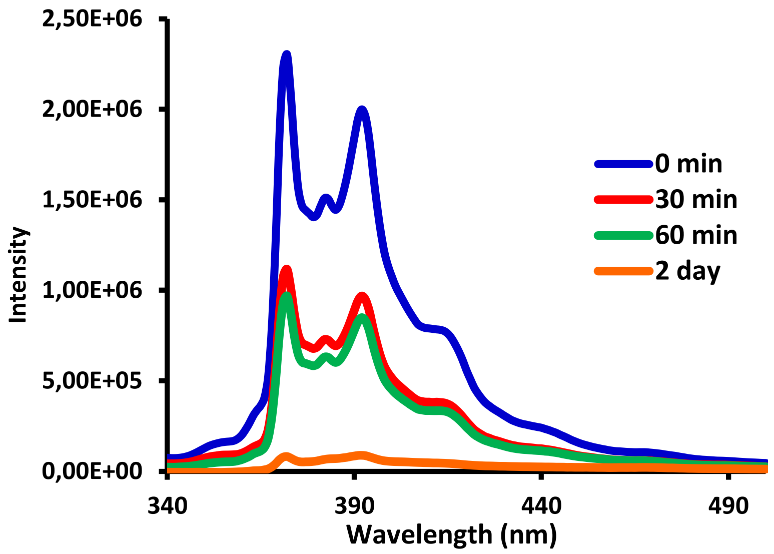

Figure S1: Sample pyrene fluorescence spectrum after exposure of dendrimer **6** at various time points.

A calibration curve was obtained to determine the quantity of pyrene in the aqueous solution at 370 nm. Fluorescence intensities values for the pyrene aliquots at 370 nm were used to determine the quantity of pyrene present in the aqueous solution.

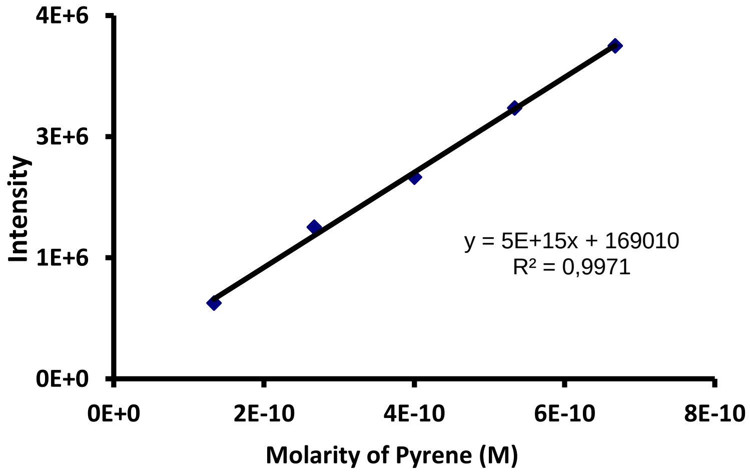

Figure S2: Calibration curve of various pyrene concentrations in a neutral, aqueous solution.

Encapsulation study results

Comparisons of the fluorescence signal at 370 nm between the dendrimers and the dendrons were graphed.

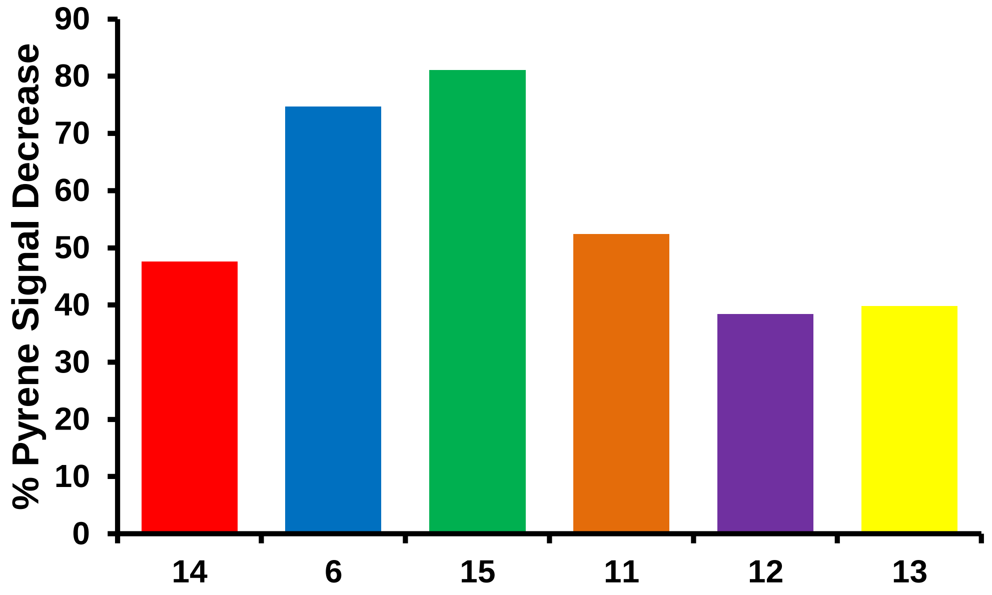

Figure S3: Percent reduction of pyrene fluorescence after 30 min exposure to the dendrimer or dendron film.

VI. Calculations of inclusion formation constants and Gibbs free energies

The following equilibrium equation was utilized for the determination of inclusion formation constants (K) and its corresponding Gibbs free energy ($\Delta G°$) [4]:

$$\text{dendrimer} + \text{pyrene} \rightleftharpoons \text{dendrimer-pyrene}$$

K = [dendrimer-pyrene]/([dendrimer][pyrene])

[dendrimer-pyrene] = the activity (concentration) of the dendrimer with the pyrene
[dendrimer] = the activity (concentration) of the dendrimer prior to exposure to pyrene
[pyrene] = the activity (concentration) of the pyrene in the aqueous solution upon exposure to the dendrimer film after 2 days.

It was assumed that the dendrimer and the dendrimer-pyrene complex exist in the solid state and therefore their values were taken to be 1. The concentration of pyrene was calculated using the pyrene fluorescence intensity at 370 nm at 2 days and the pyrene calibration curve (Figure S2). The pyrene molarity from the two trials was averaged for determination of the inclusion formation constants.

Based on the above results, the inclusion formation constant was simplified to K = 1/[pyrene].

The Gibbs free energy was determined using the following equation:
$\Delta G° = -RT\ln K$ where K = the inclusion formation constant

Table S1: Determination of inclusion constants.

Compound	average molarity (M)	1/[PAH] (M^{-1})	ΔG ($RT\ln K$)	ΔG (kcal/mol)
**14**	1.12×10^{-11}	8.92×10^{10}	-6.25×10^{4}	-14.9
**6**	4.20×10^{-11}	2.44×10^{10}	-5.93×10^{4}	-14.2
**15**	9.39×10^{-12}	1.07×10^{11}	-6.29×10^{4}	-15.0
**11**	9.79×10^{-12}	1.02×10^{11}	-6.28×10^{4}	-15.0
**12**	6.16×10^{-12}	1.62×10^{11}	-6.40×10^{4}	-15.3
**13**	1.64×10^{-10}	6.09×10^{9}	-5.58×10^{4}	-13.3

Estimation of dendrimer capacity for pyrene

Taking the aqueous solubility of pyrene to be 6.27×10^{-7} M, the capacity of dendrimer films for pyrene was calculated by determining the moles of pyrene removed from 25 mL of a saturated aqueous solution by each dendrimer film after 2 days [5].

mol pyrene removed = mol pyrene per 25 mL solution (untreated) – mol pyrene per 25 mL (treated)

Dendrimer capacity in terms of moles pyrene/ gram dendrimer was calculated as follows:

Capacity = mol pyrene removed/ mass of dendrimer film

VII. Representative 1H and 13C NMR spectral data

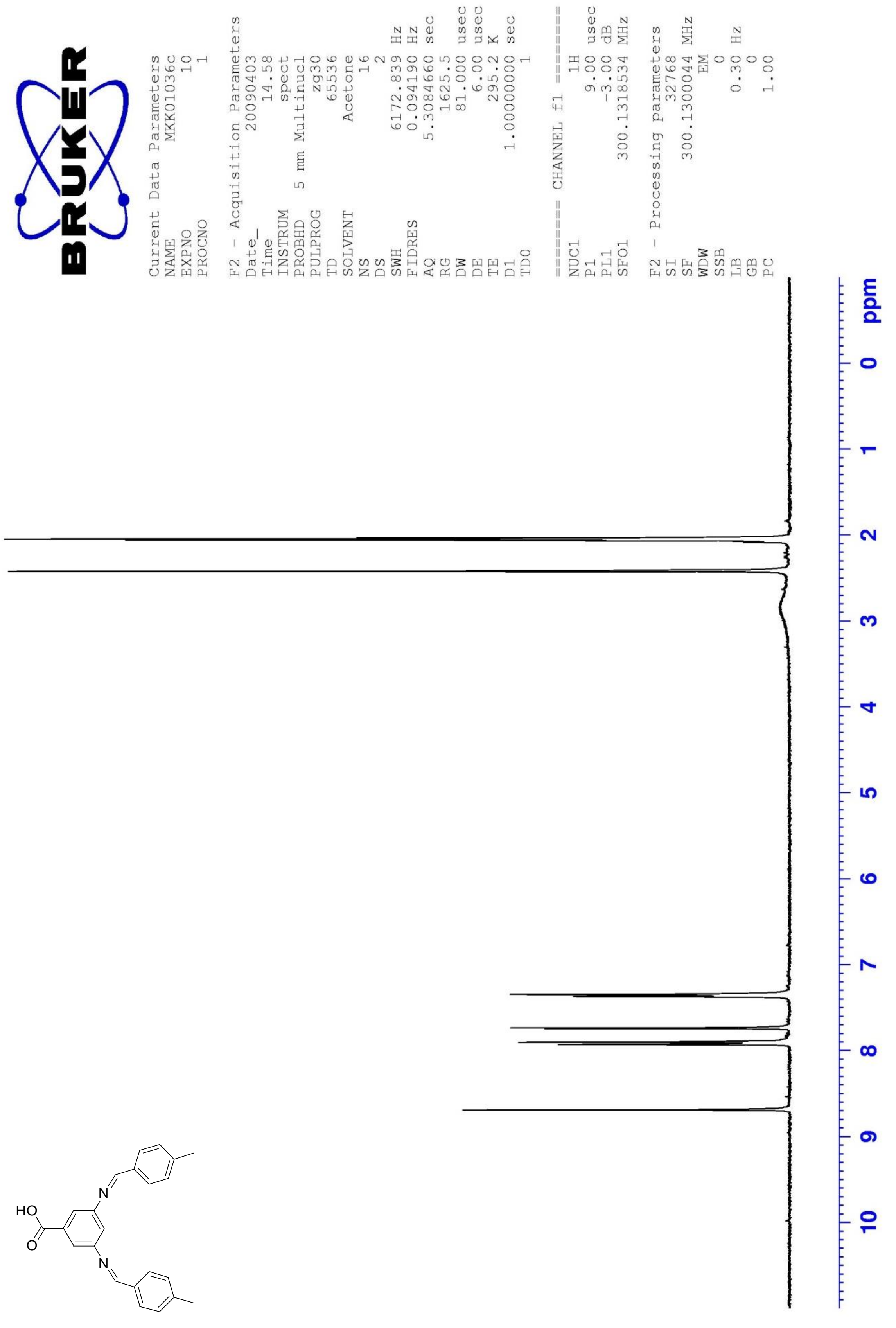

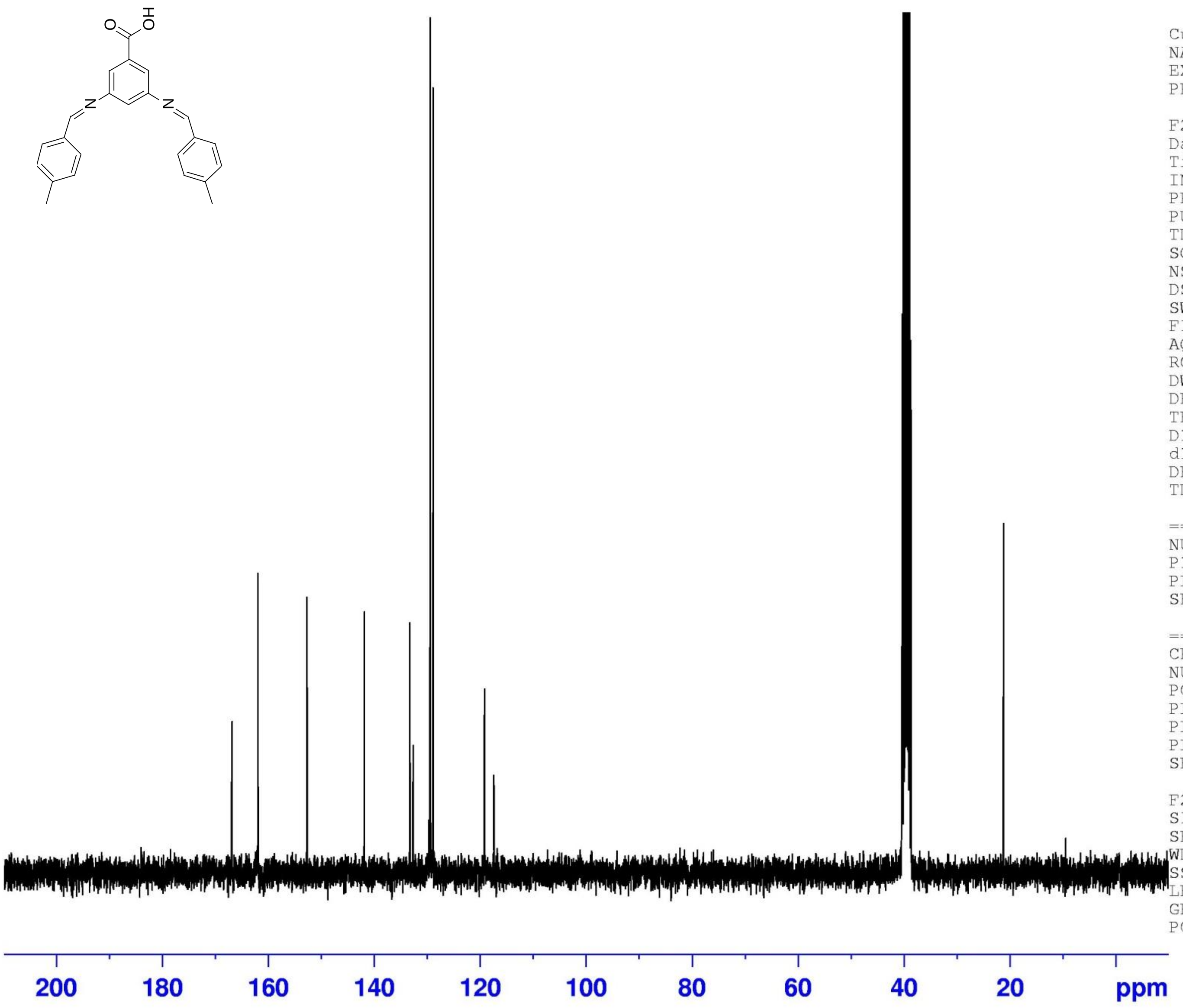


```
Current Data Parameters
NAME          mkk01045y
EXPNO                10
PROCNO                1

F2 - Acquisition Parameters
Date_          20100801
Time              13.31
INSTRUM           spect
PROBHD   5 mm Multinucl
PULPROG          zgpg30
TD                65536
SOLVENT            DMSO
NS                 1024
DS                    4
SWH           17985.611 Hz
FIDRES         0.274439 Hz
AQ            1.8219508 sec
RG               1448.2
DW               27.800 usec
DE                 6.00 usec
TE                296.2 K
D1           2.00000000 sec
d11          0.03000000 sec
DELTA        1.89999998 sec
TD0                   1

======== CHANNEL f1 ========
NUC1                13C
P1                 9.25 usec
PL1                0.00 dB
SFO1         75.4752953 MHz

======== CHANNEL f2 ========
CPDPRG2         waltz16
NUC2                 1H
PCPD2            100.00 usec
PL2               -3.00 dB
PL12              17.82 dB
PL13              17.82 dB
SFO2        300.1312005 MHz

F2 - Processing parameters
SI                32768
SF           75.4677843 MHz
WDW                  EM
SSB                   0
LB                 1.00 Hz
GB                    0
PC                 1.40
```

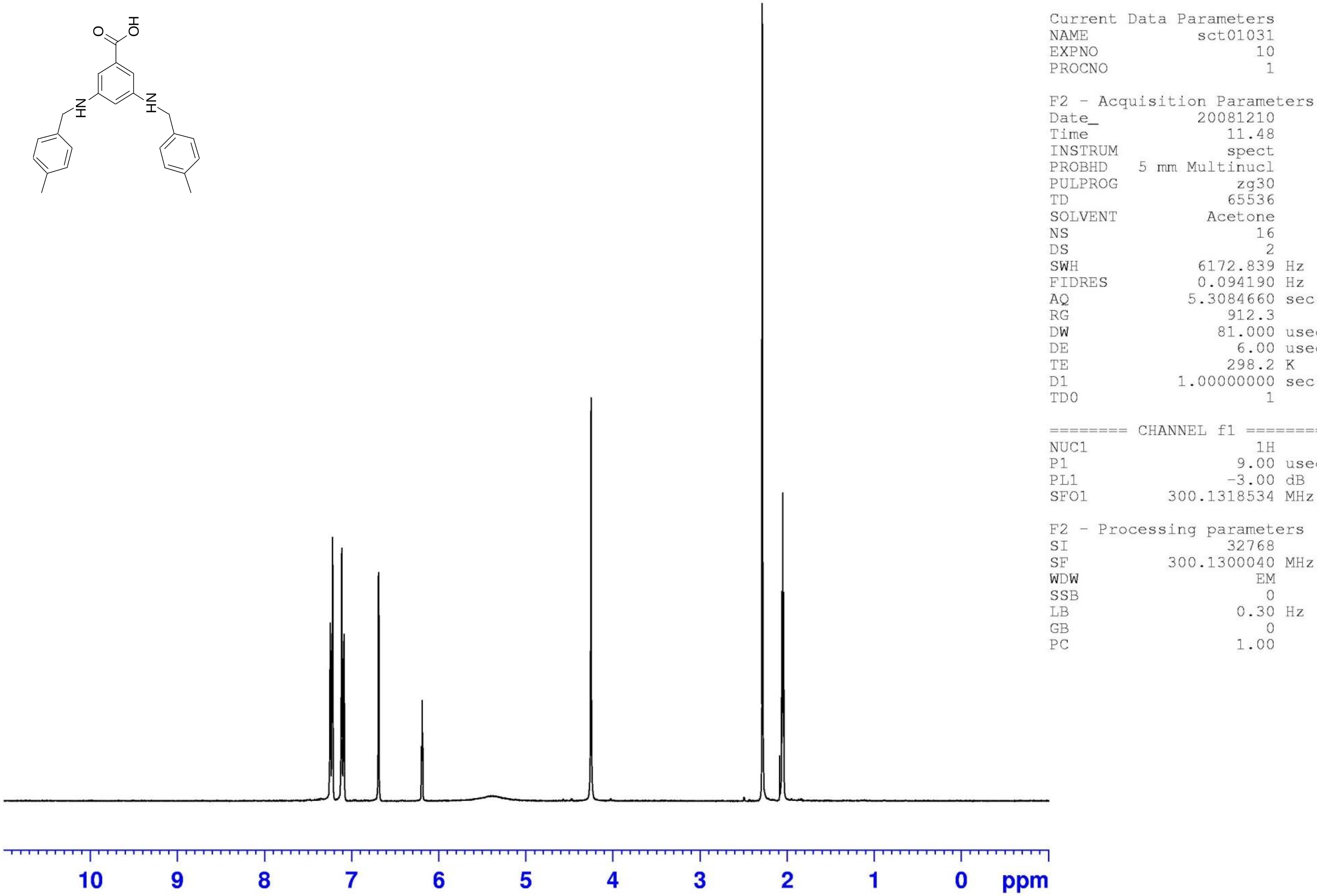
HO
O
NH
NH
10 9 8 7 6 5 4 3 2 1 0 ppm
Current Data Parameters
NAME sct01031
EXPNO 10
PROCNO 1
F2 - Acquisition Parameters
Date_ 20081210
Time 11.48
INSTRUM spect
PROBHD 5 mm Multinucl
PULPROG zg30
TD 65536
SOLVENT Acetone
NS 16
DS 2
SWH 6172.839 Hz
FIDRES 0.094190 Hz
AQ 5.3084660 sec
RG 912.3
DW 81.000 use
DE 6.00 use
TE 298.2 K
D1 1.00000000 sec
TD0 1
======== CHANNEL f1 =======
NUC1 1H
P1 9.00 use
PL1 -3.00 dB
SFO1 300.1318534 MHz
F2 - Processing parameters
SI 32768
SF 300.1300040 MHz
WDW EM
SSB 0
LB 0.30 Hz
GB 0
PC 1.00

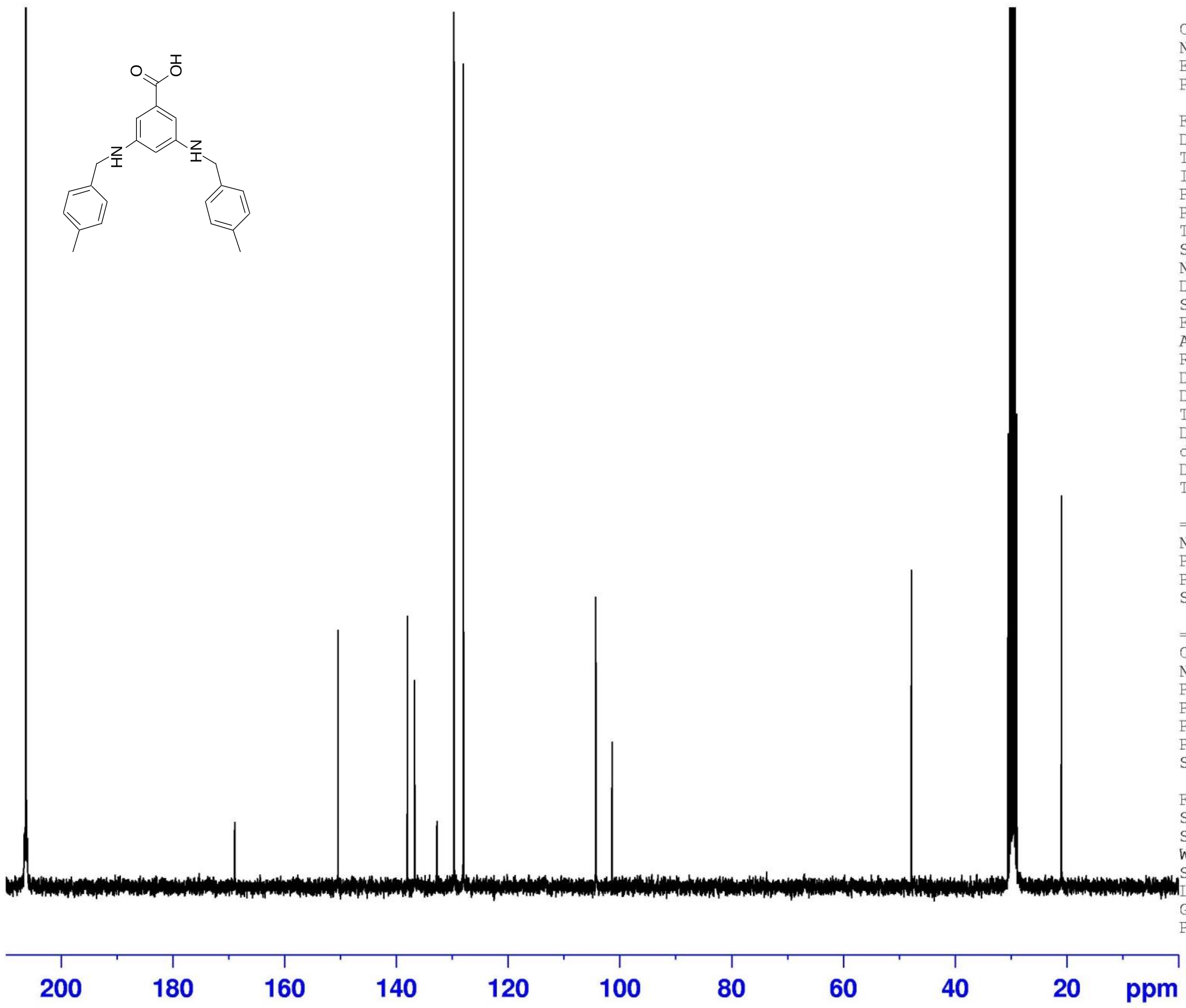


```
Current Data Parameters
NAME              SCT01031c
EXPNO                    10
PROCNO                    1

F2 - Acquisition Parameters
Date_              20090625
Time                  14.00
INSTRUM               spect
PROBHD   5 mm Multinucl
PULPROG             zgpg30
TD                    65536
SOLVENT             Acetone
NS                     1024
DS                        4
SWH               17985.611 Hz
FIDRES             0.274439 Hz
AQ                1.8219508 sec
RG                   1149.4
DW                   27.800 usec
DE                     6.00 usec
TE                    296.2 K
D1               2.00000000 sec
d11              0.03000000 sec
DELTA            1.89999998 sec
TD0                       1

======== CHANNEL f1 ========
NUC1                    13C
P1                     9.25 usec
PL1                    0.00 dB
SFO1             75.4752953 MHz

======== CHANNEL f2 ========
CPDPRG2             waltz16
NUC2                     1H
PCPD2                100.00 usec
PL2                   -2.00 dB
PL12                  19.00 dB
PL13                  19.00 dB
SFO2            300.1312005 MHz

F2 - Processing parameters
SI                    32768
SF               75.4676886 MHz
WDW                      EM
SSB                       0
LB                     1.00 Hz
GB                        0
PC                     1.40
```

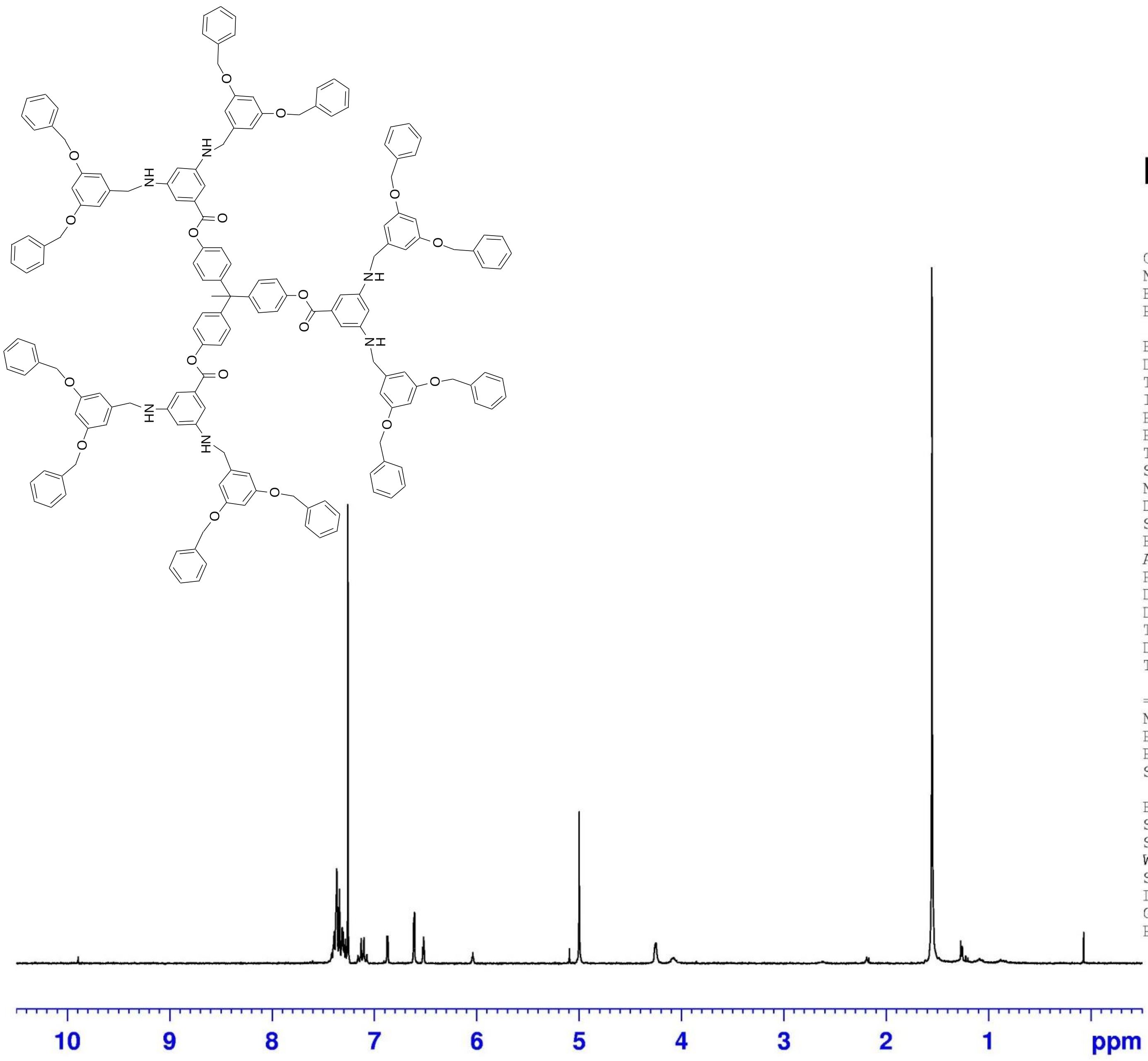

BRUKER

```
Current Data Parameters
NAME              om01053a
EXPNO                   10
PROCNO                   1

F2 - Acquisition Parameters
Date_             20110722
Time                  9.23
INSTRUM              spect
PROBHD   5 mm Multinucl
PULPROG               zg30
TD                   65536
SOLVENT              CDCl3
NS                      16
DS                       2
SWH               6172.839 Hz
FIDRES            0.094190 Hz
AQ               5.3084660 sec
RG                  1824.6
DW                  81.000 usec
DE                    6.00 usec
TE                   303.2 K
D1              1.00000000 sec
TD0                      1

======== CHANNEL f1 ========
NUC1                    1H
P1                    9.10 usec
PL1                  -3.00 dB
SFO1           300.1318534 MHz

F2 - Processing parameters
SI                   32768
SF             300.1298201 MHz
WDW                     EM
SSB                      0
LB                    0.30 Hz
GB                       0
PC                    1.00
```

VIII. References

1. Moore, J. S.; Stupp, S. I. *Macromolecules* **1990,** *23,* 65–70.

2. Percec, V.; Peterca, M.; Kowska, M. J.; Ilies, M. A.; Aqad, E.; Smidrkal, J.; Heiny, P.A. *J. Am. Chem. Soc.* **2006,** *128,* 3324–3334.

3. Cheung, S.-Y.; Chow, H. F.; Ngai, T.; Wei, X. *Chem. Eur. J.* **2009,** *15,* 2278–2288.

4. Arkas, M.; Tsiourvas, D.; Paleos, C. M. *Chem. Mater.* **2003,** *15,* 2844–2847.

5. Mackay, D.; Shiu, W. Y. *J. Chem. Eng. Data* **1977,** *22,* 399–408.
